# Erratum: Achilles' heel of triple negative cancer

**DOI:** 10.18632/oncoscience.182

**Published:** 2014-12-13

**Authors:** Alberto Ocaña, Juan C. Montero, Atanasio Pandiella

***Oncoscience. 2014; 1(2): 115-116***

***PMCID: PMC4278281 PMID: 25594005***

***Oncoscience. 2014; 1(12): 763–764***

***PMCID: PMC4303885 PMID: 25621292***

Due to the technical error the Editorial “Achilles' heel of triple negative cancer” was repeteadly published in Oncoscience, V1, issue 12, p.763-764. Original publication was published in Oncoscience, V1, issue 2, p.115-116.

